# Efficacy and Safety of Cangfu Daotan Decoction in Patients with Polycystic Ovary Syndrome: A Systematic Review and Meta-Analysis

**DOI:** 10.1155/2022/4395612

**Published:** 2022-05-17

**Authors:** Linling Wu, Han Zhang, Mengxiao Fan, Ying Yan

**Affiliations:** ^1^First Teaching Hospital of Tianjin University of Traditional Chinese Medicine, National Clinical Research Center for Chinese Medicine Acupuncture and Moxibustion, Tianjin 300381, China; ^2^Tianjin University of Traditional Chinese Medicine, Tianjin 301617, China

## Abstract

**Background:**

The infertility caused by polycystic ovary syndrome (PCOS) has received considerable attention. Considerable efforts have been made to improve the rates of pregnancy and live birth. Cangfu Daotan Decoction (CFDTD) is a classic prescription for treating infertility in obese women. The efficacy of CFDTD in PCOS is controversial.

**Objective:**

To evaluate the effectiveness and safety of CFDTD in treating infertility with PCOS.

**Methods:**

A literature search was performed in the Cochrane Library, PubMed, Embase, the China National Knowledge Infrastructure, the Wanfang Database, the VIP Chinese Biomedical science journal database, and the Chinese BioMedical database from the date of each database establishment to December 2021. Only randomized controlled trials, which were used to evaluate the efficacy of CFDTD in treating subjects with PCOS, were included in the present study. The quality of evidence was assessed using the Cochrane Reviewer Handbook 5.0.0, and meta-analysis was performed using RevMan 5.3.5 software.

**Results:**

Fourteen studies with a total of 1,433 patients were included in this analysis. The present study indicated that CFDTD could significantly improve pregnancy rate (RR = 1.62, 95% CI (1.44, 1.83), *P* < 0.00001), ovulation rate (RR = 1.40, 95% CI (1.25, 1.56), *P* < 0.00001), and estradiol levels (SMD = 0.80, 95% CI (0.03, 1.58), *P*=0.04), while testosterone levels (SMD = −0.92, 95% CI (−1.52, −0.31), *P*=0.003), homeostatic model assessment for insulin resistance (MD = −0.56, 95% CI (−0.99, −0.12), *P*=0.01), total cholesterol levels (MD = −0.60, 95% CI (−0.76, −0.44), *P* < 0.00001), triglyceride levels (MD = −0.48, 95% CI (−0.60, −0.36), *P* < 0.00001), body mass index (MD = −2.96, 95% CI (−3.88, −2.03), *P* < 0.00001), and the incidence of adverse reactions (RR = 0.47, 95% CI (0.34, 0.65), *P* < 0.00001) were significantly reduced.

**Conclusions:**

Evidence from the meta-analysis suggested that CFDTD appeared to be an effective and relatively safe treatment for PCOS. However, the influence of CFDTD on reproductive hormones, glucose metabolism, and blood lipids should be carefully concluded. Due to the low quality of the methods used in the included randomized controlled trials, further studies are required with larger sample sizes and well-designed models to confirm our findings.

## 1. Introduction

As the most common reproductive endocrine disorder, polycystic ovary syndrome (PCOS) affects approximately 7–12% of women with reproductive age [1]. PCOS not only includes symptoms of sparse menstruation, obesity, hyperandrogenemia, abnormal glucose tolerance, and hyperlipidemia but is also associated with infertility [2]. Approximately 90% of PCOS patients are infertile, and 80% of cases with ovulatory dysfunction infertility are caused by PCOS [3]. In addition, PCOS is costly, with an estimated $93 million spent annually on PCOS treatment in the United States, including an average annual cost of $533 million for infertility care [4]. Although the therapies for PCOS have been considerably optimized, the costs remain high ($669.78 per normal pregnancy) [5].

As a common cause of infertility, PCOS has attracted considerable attention [6]. At present, ovulation induction is still the mainstream treatment for PCOS infertility. Ovulation induction therapy has improved the pregnancy rate of infertility patients with PCOS; however, the efficacy of ovulation induction therapy requires further improvement [7]. Ovulation regimens include oral use of drugs, such as clomiphene and letrozole, as well as in vitro fertilization (IVF) [8]. However, neither clomiphene nor letrozole can completely eliminate the problem of infertility (live birth rate clomiphene 25.2%, letrozole 30.2–37.6%) [9]. The improvement of the effectiveness of ovulation induction therapy is imperative to reduce the incidence of infertility. Ovulation induction can also cause side effects, including luteinized unruptured follicle (LUF) and ovarian hyperstimulation syndrome [10]. The majority of the patients with infertility exhibit complications such as insulin resistance and hyperandrogenemia. Previous studies have shown that the effect of clomiphene is affected by the serum levels of insulin, and the improvement of insulin resistance can increase clinical pregnancy and possibly shorten the duration of pregnancy [11, 12]. Similarly, the efficacy of letrozole is influenced by the total serum testosterone levels. Therefore, it is imperative to identify a safe, effective, and multitargeted drug to regulate the reproduction and metabolism of PCOS as an adjuvant therapy.

In the past 50 years, the use of complementary and alternative medicine and the study of Cangfu Daotan Decoction (CFDTD) for PCOS have been gradually increased [13, 14]. In China, CFDTD was first reported to be used in the mid-16th century in Guangsi Ji Yao. According to this book, CFDTD was used for treating infertility in obese women [15]. In addition, CFDTD can also be used in obese women with oligomenorrhea. Since no records of PCOS have been reported in ancient China, ancient books have been used for prescriptions based on the typical symptoms of PCOS, including irregular menstruation, obesity, and infertility. In traditional medicine, CFDTD has been used to treat the phlegm-dampness syndrome, which is very similar to the typical symptoms of obese PCOS, including obesity, irregular menstruation, and acanthosis nigricans. An accumulated number of studies have shown that CFDTD can be used to treat PCOS, notably in obese PCOS cases [16–18]. Concomitantly, a high number of studies have been conducted to explore the effect and mechanism of CFDTD. These studies have suggested that CFDTD can improve infertility by improving insulin resistance, reducing hyperlipidemia, hyperandrogenemia, and body weight [19–22]. The research on CFDTD is not consistent. Zhao et al. [23] suggested that CFDTD did not significantly increase the pregnancy rate compared with that of the placebo. Similar conclusions were reported in a previous study by Chen et al. which suggested that CFDTD did not significantly improve the ovulation rate [24]. However, other studies have shown that CFDTD can significantly increase pregnancy and ovulation rates [25–27]. Moreover, the conclusions drawn regarding the safety of CFDTD are contradictory [28, 29]. Therefore, a meta-analysis was performed to systematically review and evaluate the efficacy and safety of CFDTD as an adjunctive drug for PCOS.

## 2. Materials and Methods

### 2.1. Search Strategy

This meta-analysis was performed according to the Preferred Reporting Items for Systematic Reviews and Meta-Analyses (PRISMA) and the Cochrane Handbook for Systematic Reviews of Interventions.

### 2.2. Inclusion and Exclusion Criteria

#### 2.2.1. Type of Study

A randomized controlled trial (RCT) study design was used, which was based on the treatment of CFDTD in PCOS.

#### 2.2.2. Objective of the Study

The patients were diagnosed with PCOS according to the Rotterdam conference recommendations of 2003. There was no restriction on age, race, or region of study subjects.

#### 2.2.3. Intervention Measures

CFDTD alone or CFDTD combined with Western medicine or CFDTD combined with complementary and alternative medical therapies (including drugs, acupuncture, exercise, and diet change) was used. There was no limit to the dosage and course of CFDTD. The control group included blank control, placebo control, Western medicine, and complementary and alternative medicine therapy groups.

#### 2.2.4. Outcome Indicators

The primary outcome measured was the pregnancy rate; the secondary outcomes were the following: rate of ovulation, reproductive hormone levels (including follicle-stimulating hormone (FSH), luteinizing hormone (LH), estradiol (*E*_2_), and testosterone (*T*) levels), assessment of glucose metabolism (homeostatic model assessment insulin resistance (HOMA-IR)), assessment of lipid metabolism (including total cholesterol (TC), triglyceride (TG), and high-density lipoprotein (HDL) levels, as well as low-density lipoprotein (LDL) concentration), body mass index (BMI), and the gestational safety as the incidence of adverse events.

This systematic evaluation was used to explore the efficacy of CFDTD in treating subjects with PCOS. Therefore, only the evaluation results of each study at the end of the course of treatment were extracted, without considering the periodic evaluation results.

#### 2.2.5. Exclusion Criteria

The following exclusion criteria were used: (1) literature that contained incomplete data and could not be analyzed, (2) literature, which was published repeatedly, (3) subjects with PCOS and other diseases or serious complications, (4) subjects of the experimental and the control groups treated with CFDTD and lack of the aim of the study to verify the effectiveness of CFDTD, and (5) the addition of CFDTD and the reduced prescription for the study intervention measures were not included.

### 2.3. Literature Retrieval Strategy

The following databases were searched: Cochrane Library, PubMed, Embase, VIP Chinese Biomedical science journal database, the China National Knowledge Infrastructure (CNKI), WanFang Data databases, and the Chinese BioMedical database (CBM) from the date of inception to December 31, 2021. Only RCTs, which were used to evaluate the efficacy of CFDTD in PCOS, were included in the present study. The keywords and free words were used to search. The English search terms included the following: Polycystic Ovary Syndrome, Polycystic Ovarian Syndrome, PCOS, cangfu daotan, cangfu daotan, cang fu dao tan, randomized, randomly, randomized controlled trial, and clinical trial. For example, Table 1 is the specific retrieval strategy used in PubMed. To avoid missing information, a manual search of the references to similar studies was conducted.

### 2.4. Literature Selection, Data Extraction, and Bias Risk Assessment of the Included Studies

Literature screening and data extraction were independently completed by two researchers and cross-checked. Any discrepancy was solved by consultation with a third reviewer. A form was created for data extraction, which included the summary of general data (including title, author, research location, and publication time), the basic characteristics of the study (including the number of cases, age, and intervention measures of the experimental group and the control group), the key elements of risk assessment for bias, the outcome indicators, and the outcome measurement data. Cochrane systematic assessor manual 5.3.0 was used as a risk assessment tool for RCTs to evaluate the risk of bias in the included studies.

### 2.5. Statistical Analysis

The RevMan 5.3 software was used for meta-analysis. The relative risk was used as the effect index for the enumeration data. Mean difference (MD) or standardized mean difference (SMD) was used as the effect index for measurement data. The confidence interval was set at 95%. The *χ*^2^ test was used to analyze significant differences between the results, and *P* < 0.05 was considered to indicate a statistically significant difference. The heterogeneity was quantitatively assessed by *I*^2^. In the absence of statistical heterogeneity among the results, the fixed effect model was used for meta-analysis. In case statistical heterogeneity was noted among the results of each study, the source of heterogeneity was analyzed, and the influence of significant clinical heterogeneity was excluded. Finally, a random-effect model was used for meta-analysis. Significant clinical heterogeneity was treated with subgroup analysis or sensitivity analysis or only descriptive analysis.

## 3. Results

### 3.1. Study Characteristics

**Figure 1:**
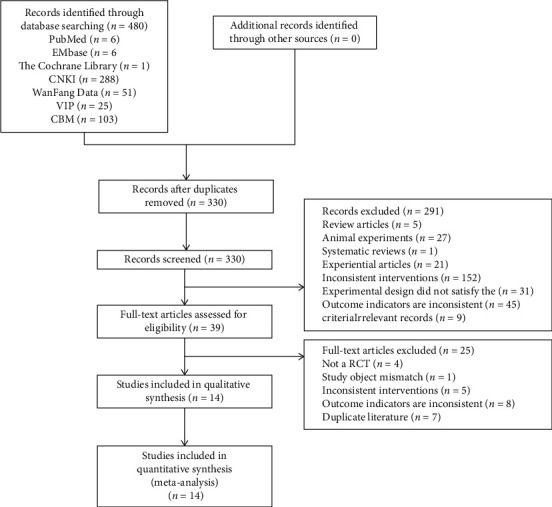
Flow diagram of the study selection process.

The search strategy identified 480 studies. A total of 466 publications were excluded due to the repetition of data or due to being irrelevant or nonspecific to the study topic. This classification was performed based on the title, abstract, and full text. Finally, 14 studies with a total of 1,433 patients were included in the quantitative analyses (Figure 1).

### 3.2. Basic Characteristics and Bias Risk Assessment of the Included Studies

**Figure 2:**
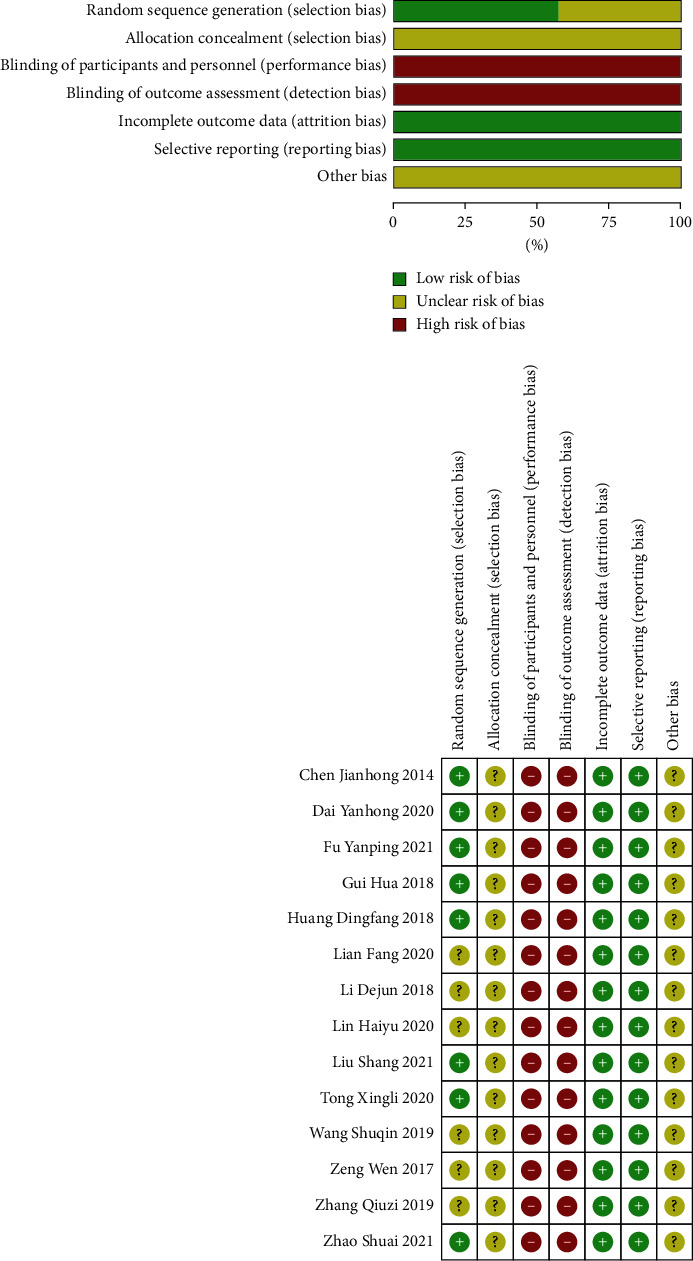
The risk of bias of the included studies.

All included studies indicated no significant differences at baseline between the experimental and treatment groups. Eight studies reported randomization using a random number table, while the other six studies did not report specific methods for achieving randomization. None of the 14 trials described allocation hiding. Only one study included a blind study design; however, following the reading of its full text, it was found to be falsely blinded. None of the 14 studies mentioned withdrawal or follow-up. Due to the lack of detail and specific information, it was impossible to determine whether sufficient implementation had taken place during random sequence generation, blinding, or allocation hiding. As a result, the trials included in the present study were of poor quality (Figure 2). The basic characteristics of the included studies are shown in Table 2.

### 3.3. Outcomes

#### 3.3.1. Pregnancy Rate

**Figure 3:**
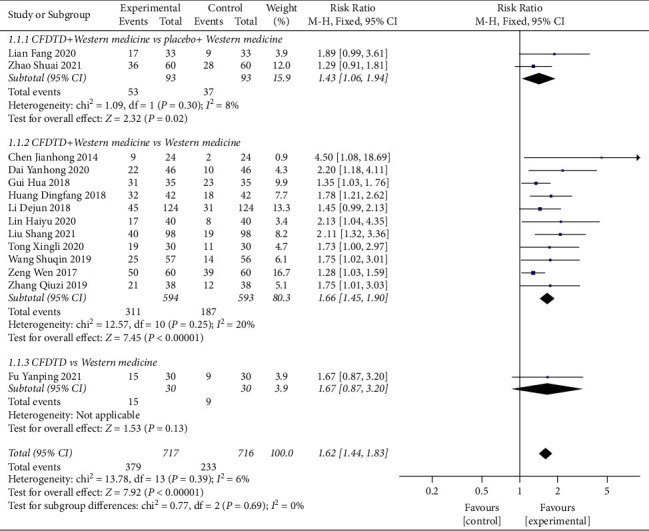
Meta-analyses of the effect of CFDTD on pregnancy rate.

**Figure 4:**
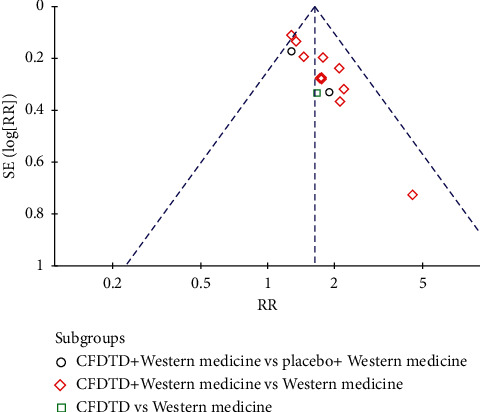
Funnel plot of pregnancy rate for the publication bias.

A total of 14 studies including 1,433 patients (717 in the experimental and 716 in the control groups, respectively) assessed pregnancy rates [23–36]. The literature indicated homogeneity (heterogeneity test severity *χ*^2^ = 13.78, *P*=0.39, *I*^2^ = 6%), and the combined effect amount adopted the fixed effect model. The analysis indicated that CFDTD could significantly increase the pregnancy rate of PCOS (RR = 1.62, 95% CI (1.44, 1.83), *P* < 0.00001) (Figure 3). Funnel plot was used to estimate publication bias, and the results demonstrated no significant publication bias (Figure 4).

A subgroup analysis was performed for the included trials based on different interventions. Two RCTs [23, 33] compared the efficacy of CFDTD + Western medicine with placebo + Western medicine on the pregnancy rate. The literature indicated homogeneity (heterogeneity test severity *χ*^2^ = 1.09, *P*=0.30, *I*^2^ = 8%), and the combined effect adopted the fixed effect model. The results indicated that CFDTD + Western medicine exhibited a significant effect on improving pregnancy rate than that of placebo + Western medicine in the PCOS subjects (RR = 1.43, 95% CI (1.06, 1.94), *P*=0.02) (Figure 3.1.1.1).

A total of 11 RCTs [24–30, 32, 34–36] compared the efficacy between CFDTD + Western medicine with Western medicine on the pregnancy rate. The literature indicated homogeneity (heterogeneity test severity *χ*^2^ = 12.57, *P*=0.25, *I*^2^ = 20%), and the combined effect followed the fixed effect model. The results indicated that CFDTD + Western medicine used for PCOS was more effective than Western medicine used to improve pregnancy rate (RR = 1.66, 95% CI (1.45, 1.90), *P* < 0.00001) (Figure 3.1.1.2).

Only one RCT [31] reported the incidence of the pregnancy rate in CFDTD compared with that noted in Western medicine. Meta-analysis indicated that CFDTD increased the pregnancy rate of PCOS compared with that noted following Western medicine (RR = 1.67, 95% CI (0.87, 3.20), *P*=0.13); however, these results were not statistically significant (Figure 3.1.1.3).

#### 3.3.2. Ovulation Rate

**Figure 5:**
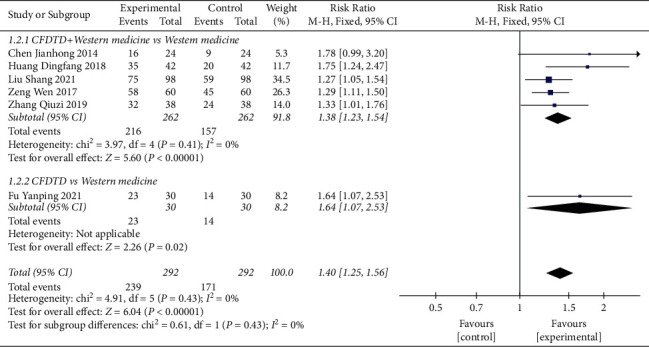
Meta-analyses of the effect of CFDTD on the ovulation rate.

The ovulation rate was assessed in 584 patients (experimental group 292, control group 292) derived from six studies [24–27, 31, 36]. The literature was homogenous (heterogeneity test Chi ^2^ = 4.91, *P*=0.43, *I*^2^ = 0%), and the combined effect size followed the fixed effect model. CFDTD significantly increased the ovulation rate of PCOS compared with that noted in the control group (RR = 1.40, 95% CI (1.25, 1.56), *P* < 0.00001) (Figure 5).

A subgroup analysis was also performed in the included trials based on interventions. Five RCTs [24–27, 36] compared the effects of CFDTD + Western medicine with Western medicine on the ovulation rate. Heterogeneity was not significant in the literature (heterogeneity test Chi^2^ = 3.97, *P*=0.41, *I*^2^ = 0%), and the fixed-effect model was adopted. Compared with Western medicine, CFDTD + Western medicine significantly increased the ovulation rate of PCOS subjects (RR = 1.38, 95% CI (1.23, 1.54), *P* < 0.00001) (Figure 5.1.2.1).

Only one trial [31] compared the efficacy of CFDTD with that of Western medicine. The meta-analysis results indicated that CFDTD significantly increased the ovulation rate of PCOS compared with that of the Western medicine group (RR = 1.64, 95% CI (1.07, 2.53), *P*=0.02) (Figure 5.1.2.2).

#### 3.3.3. Reproductive Hormones

**Figure 6:**
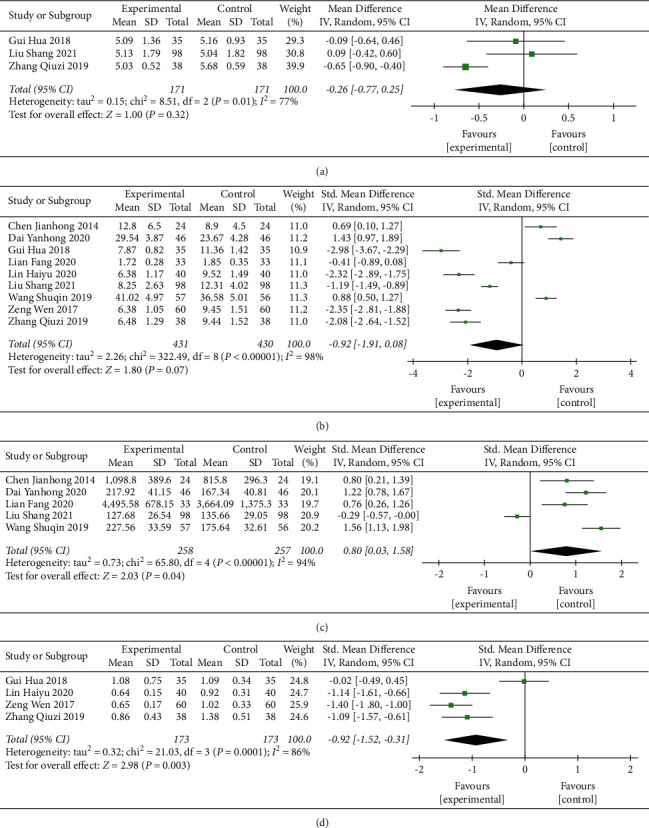
Meta-analyses of the effect of CFDTD on reproductive hormones: (a) FSH; (b) LH; (c) *E*_2_; (d) *T*.

Three studies evaluated the influence of CFDTD on FSH [26, 32, 36]. The heterogeneity of the literature was statistically significant (heterogeneity test Chi ^2^ = 8.51, *P*=0.01, *I*^2^ = 77%), and the random effect model was used. The results indicated that CFDTD treatment did not cause significant changes in FSH levels in PCOS compared with those noted in the control group (MD = −0.26, 95% CI (−0.77, 0.25), *P*=0.32) (Figure 6(a)). Due to the large heterogeneity, sensitivity analysis was performed. We excluded Zhang Qiuzi 2019 [36], which indicated minimal heterogeneity (heterogeneity test *χ*^2^ = 0.22, *P*=0.64, *I*^2^ = 0%). The combined effect test indicated no significant difference between the groups (MD = 0.01, 95%CI (−0.36, 0.38), *P*=0.97). Sensitivity analysis indicated no statistical differences in FSH levels between the two groups.

A total of 9 studies [24, 26, 27, 29, 30, 32–34, 36] evaluated the impact of CFDTD on LH levels. The literature did not contain homogenous data (heterogeneity test *χ*^2^ = 322.49, *P* < 0.00001, *I*^2^ = 98%), and the effect size was combined by the random effect model. The results indicated that CFDTD exhibited no significant differences in the treatment of LH in PCOS (SMD = −0.92, 95% CI (−1.91, 0.08), *P*=0.07) (Figure 6(b)). Due to the large heterogeneity, a sensitivity analysis was performed. Following the removal of the study by Dai Yanhong 2020 [30], the heterogeneity was still large (heterogeneity test *χ*^2^ = 228.03, *P* < 0.00001, *I*^2^ = 97%); however, the combined effect test indicated significant differences between the groups (SMD = −1.21, 95% CI (−2.17, −0.25), *P*=0.01). Sensitivity analysis indicated that compared with the control group, the effect of CFDTD on LH levels was not consistent.

A total of 5 studies [24, 26, 29, 30, 33] evaluated the effect of CFDTD on *E*_2_ levels. The literature did not reveal homogeneity (heterogeneity test *χ*^2^ = 65.80, *P* < 0.00001, *I*^2^ = 94%), and the combined effect size followed the random effect model. The results indicated that CFDTD could significantly increase the *E*_2_ levels of PCOS (SMD = 0.80, 95% CI (0.03, 1.58), *P*=0.04) (Figure 6(c)). Due to the large heterogeneity present, sensitivity analysis was performed. Following the exclusion of two articles [26, 29], it was shown that heterogeneity was small (heterogeneity test *χ*^2^ = 2.23, *P*=0.33, *I*^2^ = 10%). The combined effect test indicated that the difference between the two groups was statistically significant (SMD = 0.96, 95% CI (0.65, 1.27), *P* < 0.00001). The sensitivity analysis indicated that compared with the control group, CFDTD could significantly increase *E*_2_ levels; in addition, the results were consistent.

A total of 4 studies [27, 32, 34, 36] evaluated the influence of CFDTD on the *T* levels. The literature did not contain homogenous data (heterogeneity test *χ*^2^ = 21.03, *P*=0.0001, *I*^2^ = 86%), and the effect size followed the random effect model. The results indicated that CFDTD could significantly reduce the *T* levels of the PCOS subjects (SMD = −0.92, 95%CI (−1.52, −0.31), *P*=0.003) (Figure 6(d)). Due to the large heterogeneity, sensitivity analysis was performed. The study by Gui Hua et al. in 2018 was excluded [32], and the heterogeneity was small (heterogeneity test *χ*^2^ = 1.15, *P*=0.56, *I*^2^ = 0%). The combined effect test indicated significant differences between the two groups (SMD = −1.23, 95% CI (−1.49, −0.98), *P* < 0.00001). Sensitivity analysis indicated that CFDTD could significantly reduce *T* levels; the results were consistent.

#### 3.3.4. Glucose Metabolism

**Figure 7:**
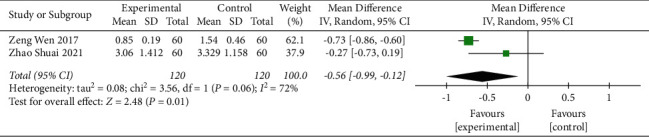
Meta-analyses of the effect of CFDTD on HOMA-IR.

Two studies [23, 27] evaluated the influence of CFDTD on HOMA-IR. The heterogeneity of the literature was statistically significant (heterogeneity test *χ*^2^ = 3.56, *P*=0.06, *I*^2^ = 72%), and the random effect model was used. The results suggested that CFDTD could significantly reduce HOMA-IR levels of PCOS subjects (MD = −0.56, 95%CI (−0.99, −0.12), *P*=0.01) (Figure 7).

#### 3.3.5. Lipid Metabolism

Only one trial investigated the effect of CFDTD on blood lipid levels [35]. Meta-analysis indicated that CFDTD significantly reduced TC (MD = −0.60, 95% CI (−0.76, −0.44), *P* < 0.00001) and TG (MD = −0.48, 95% CI (−0.60, −0.36), *P* < 0.00001) levels. No significant differences were noted in LDL and HDL levels between the two groups.

#### 3.3.6. BMI

**Figure 8:**
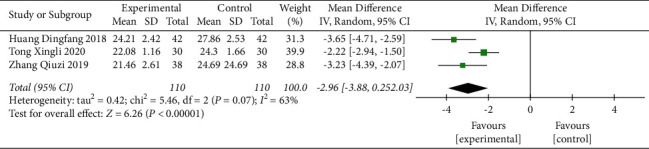
Meta-analyses of the effect of CFDTD on BMI.

Three studies [25, 35, 36] evaluated the influence of CFDTD + Western medicine compared with that of Western medicine on BMI. The heterogeneity of the literature was statistically significant (Chi^2^ = 5.46, *P*=0.07, *I*^2^ = 63%), and the random effect model was used (MD = −2.96, 95% CI (−3.88, −2.03), *P* < 0.00001). The results suggested that CFDTD + Western medicine could significantly reduce the BMI of PCOS subjects (Figure 8).

#### 3.3.7. Incidence of Adverse Reactions

**Figure 9:**
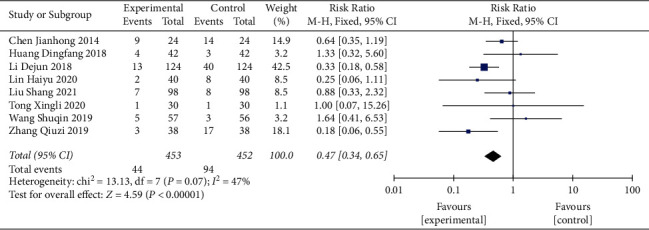
Meta-analyses of the effect of CFDTD on the incidence of adverse reactions.

Nine studies reported adverse reactions [24–26, 28, 29, 31, 34–36]. The incidence of adverse reactions between CFDTD + Western medicine and Western medicine was compared in eight RCTs. The literature exhibited homogeneity (heterogeneity test *χ*^2^ = 13.13, *P*=0.07, *I*^2^ = 47%), and the fixed-effect model was adopted. The results indicated that CFDTD + Western medicine significantly reduced the incidence of adverse reactions in PCOS subjects compared with that of the Western medicine treatment group (RR = 0.47, 95% CI (0.34, 0.65), *P* < 0.00001) (Figure 9). In addition, a comparison between CFDTD and Western medicine was reported in one trial, and no adverse reactions occurred in both groups [31].

## 4. Discussion

In the present study, the differences in reproduction (pregnancy rate and ovulation rate), metabolism (reproductive hormones, glucose metabolism, and lipid metabolism), and safety of PCOS between CFDTD and different intervention programs were assessed. A total of 1,433 people were included in 14 studies. The function of CFDTD was summarized as follows: removing dampness, reducing phlegm, and strengthening the spleen. It was composed of the following constituents: *Rhizoma atractylodis*, *sweet attached*, *tangerine peel*, *Bile south star*, *Hovenia dulcis*, *pinellia*, *Rhizoma ligustici wallichii*, *talc*, *Poria cocos*, and *medicated leaven. Rhizoma atractylodis* can clear damp phlegm, and its function is to reduce blood glucose levels and the development of obesity [37]. Previous studies have shown that the reduction in the blood lipid levels is an important effect of *tangerine peel* and *Poria cocos* [38, 39]. *Sweet attached, Hovenia dulcis*, and *pinellia* can improve gastrointestinal function [40–42]. CFDTD has been used to treat infertility in ancient China, and modern studies have also confirmed this effect. Network pharmacological analysis suggested that the combination of *Rhizoma atractylodis*, *sweet attached*, and *pinellia* could improve insulin resistance, reduce the levels of inflammatory cytokines, regulate endocrine hormones and the reproductive axis, promote follicular maturation and ovulation, and ultimately improve reproductive ability [43]. The results of this meta-analysis demonstrated that CFDTD could significantly improve the pregnancy rate of PCOS. Subgroup analysis indicated that CFDTD significantly increased the pregnancy rate of PCOS subjects compared with that of the placebo and the blank control group. Compared with Western medicine, CFDTD did not exhibit a statistically significant effect on the pregnancy rate. In terms of the ovulation rate, CFDTD has also been shown to significantly increase the ovulation rate. The exclusion of individual studies did not significantly affect the overall effect size of the pregnancy and ovulation rates. This confirmed that CFDTD was effective in improving the reproduction of PCOS subjects (notably pregnancy and ovulation rates), and the results were consistent. Regrettably, live birth rates were not reported in the included studies.

Moreover, the present study evaluated the effects of CFDTD on the levels of reproductive hormones, glucose metabolism, lipid metabolism, and BMI. With regard to the investigation of reproductive hormones, CFDTD exhibited no significant effect on FSH and LH levels, whereas it significantly increased *E*_2_ levels and decreased *T* levels. However, the results indicated significant heterogeneity. By using sensitivity analysis, it was found that CFDTD exhibited consistent results on FSH, *E*_2_, and *T* levels. However, the effect size of CFDTD on LH levels was altered following the removal of specific literature studies, and the results were not consistent. Therefore, this conclusion should be treated with caution. Modern medicine has also confirmed that the improvement in the concentration levels of the reproductive hormones alleviates ovulation disorders in PCOS. Hyperandrogenemia is an endocrine characteristic of PCOS. High androgen levels can inhibit *E*_2_ levels, and low *E*_2_ levels can lead to excessive secretion of LH by the pituitary gland. Combined with the negative feedback produced by estrogen, FSH secretion levels are significantly reduced [44]. Due to high levels of androgens and reduced FSH levels, the follicles in PCOS patients stop developing to a certain state, which leads to disorders in follicle maturation and ovulation [45]. Therefore, CFDTD improves abnormal levels of reproductive hormones in PCOS patients by increasing *E*_2_ levels and decreasing *T* levels, which ultimately improves ovulation disorders.

In the present study, it was also found that CFDTD could improve abnormal glucose tolerance and reduce blood lipids and weight gain. However, the CFDTD group exhibited large heterogeneity in HOMA-IR and BMI. It can also be deduced from the figure that the results of each study reflected the superiority of the experimental group compared with the control group. Since the effect size of each study was different, certain studies were decreased to a higher and a lower extent, which led to the emergence of heterogeneity. Therefore, a sensitivity analysis was not performed on the study group. CFDTD has been applied to patients with phlegm-dampness syndrome and obesity as reported by early studies [15]. Recent studies have confirmed the effects of both obesity and glucose and lipid metabolism on fertility [21, 46]. It has been shown [47, 48] that a 5% decrease in body weight of PCOS patients can reduce fasting blood glucose and serum insulin levels, thereby improving IR. Concomitantly, the reduction in the body weight of the PCOS patients can reduce abdominal fat, decrease serum TG and LDL levels, and increase HDL levels [2]. In addition, weight loss can reduce leptin levels in the body, which inhibits the secretion of ovarian steroids regulated by the neuroendocrine system, ultimately improving the menstrual cycle, promoting ovulation, and increasing the chance of pregnancy. However, in the present meta-analysis, only one study was included that evaluated the effects of CFDTD on blood lipid levels. Therefore, the conclusions regarding the effects of CFDTD on blood lipids should be drawn with caution.

In addition, nine studies mentioned adverse effects. Although these adverse reactions were mild and resolved quickly, the results of the present study indicated that CFDTD significantly reduced the incidence of adverse reactions. This indicates that the safety margin of CFDTD was optimal.

The present study contains certain limitations as follows: (1) a certain risk of bias was noted in the included literature, and the majority of the studies were not fully described in terms of allocation hiding, blind implementation, and selectivity of the research results. Certain risks of selectivity and implementation bias were evident. (2) The sample size of the included literature was small, and the effectiveness of the test was limited. (3) Certain intervention measures were noted in different studies resulting in various confounding factors. Heterogeneity was apparent among studies. (4) All studies were conducted in China and published in domestic journals, which may lead to bias. The aforementioned limitations may reduce the reliability of the meta-analysis. Finally, a larger sample size is required and a multicenter study design to further validate the effectiveness of CFDTD in the treatment of PCOS.

## 5. Conclusion

In conclusion, the systematic evaluation of CFDTD for the treatment of PCOS indicated that the CFDTD was beneficial to the pregnancy and ovulation rates, while its significance was not clear on the reproductive hormones, glucose metabolism, lipid metabolism, and BMI. In addition, the results of the present study suggested that CFDTD was generally safe. However, due to the low quality of the studies included, we recommend caution in generalizing these results. In the future, additional large-sample, multicenter prospective studies are required to obtain scientific, objective, and reliable conclusions.

## Figures and Tables

**Table 1:** Literature retrieval strategy of PubMed.

#1 “polycystic ovary syndrome” [MeSH Terms] OR “polycystic ovary syndrome” [Title/Abstract] OR “polycystic ovarian syndrome” [Title/Abstract] OR “PCOS” [Title/Abstract]

#2 “Cangfudaotan” [Title/Abstract] OR “Cangfu daotan” [Title/Abstract] OR “cang fu dao tan” [Title/Abstract]

#3 “randomized controlled trials as topic” [MeSH terms] OR “randomized controlled trial” [Title/Abstract] OR “randomized” [Title/Abstract] OR “randomly” [Title/Abstract] OR “clinical trial” [Title/Abstract]

**Table 2:** The details of the included studies.

Studies	Study location	Sample size		Age (T/C)	BMI (kg/m2) (T/C)	Intervention		Course of treatment	Outcomes
		T	C			T	C		
Zhao Shuai 2021	Shandong province, China	60	60	32.22 ± 4.35/30.80 ± 4.69	28.05 ± 1.88/28.63 ± 2.78	CFDTD + Western medicine	Placebo + Western medicine	21 days	①④
Lian Fang 2020	Shandong province, China	33	33	30.00 ± 3.07/29.70 ± 3.74	—	CFDTD + Western medicine	Placebo + Western medicine	21 days	①③
Liu Shang 2021	Zhejiang province, China	98	98	27.60 ± 3.50/27.80 ± 3.30	—	CFDTD + Western medicine	Western medicine	3 months	①②③⑥
Lin Haiyu 2020	Shandong province, China	40	40	31.19 ± 5.42/31.04 ± 5.31	—	CFDTD + Western medicine	Western medicine	30 days	①③⑥
Huang Dingfang 2018	Jiangxi province, China	42	42	24.87 ± 4.67/24.59 ± 4.92	32.20 ± 4.23/31.32 ± 4.65	CFDTD + Western medicine	Western medicine	3 months	①②⑥
Zeng Wen 2017	Hunan province, China	60	60	32.35 ± 5.64/32.23 ± 5.60	—	CFDTD + Western medicine	Western medicine	4 months	①②③④
Chen Jianhong 2014	Zhejiang province, China	24	24	28.50 ± 8.90/27.20 ± 8.50	—	CFDTD + Western medicine	Western medicine	6 months	①②③⑥
Dai Yanhong 2020	Henan province, China	46	46	29.62 ± 3.57/29.84 ± 3.32	—	CFDTD + Western medicine	Western medicine	4 months	①③
Gui Hua 2018	Zhejiang province, China	35	35	28.12 ± 2.23/27.56 ± 2.31	22.24 ± 1.18/22.15 ± 1.24	CFDTD + Western medicine	Western medicine	4 months	①③
Li Dejun 2018	Xinjiang Uygur autonomous region, China	124	124	25.64 ± 1.30/26.17 ± 1.82	—	CFDTD + Western medicine	Western medicine	3 months	①⑥
Zhang Qiuzi 2019	Fujian province, China	38	38	27.50 ± 3.60/28.40 ± 5.60	25.70 ± 2.52/25.02 ± 2.96	CFDTD + Western medicine	Western medicine	3 months	①②③⑥
Wang Shuqin 2019	Henan province, China	57	56	31.46 ± 4.43/31.51 ± 4.40	22.18 ± 1.02/22.43 ± 1.01	CFDTD + Western medicine	Western medicine	3 months	①③⑥
Tong Xingli 2020	Jiangsu province, China	30	30	28.80 ± 3.43/29.57 ± 2.61	26.50 ± 1.14/27.23 ± 1.83	CFDTD + Western medicine	Western medicine	3 months	①⑤⑥
Fu Yanping 2021	Hunan province, China	30	30	27.87 ± 3.93/28.07 ± 3.81	—	CFDTD	Western medicine	3 months	①②⑥

T: Treatment group; C: Control group; —: Unreported; ①: Pregnancy rate; ②: Ovulation rate; ③: Reproductive Hormones; ④: Glucose Metabolism; ⑤: Lipid Metabolism; ⑥: Incidence of adverse reactions; CFDTD: Cangfu Daotan Decoction.

## Data Availability

The data in this study can be obtained from the corresponding author.
